# Role of Glyoxalase 1 (*Glo1*) and methylglyoxal (MG) in behavior: recent advances and mechanistic insights

**DOI:** 10.3389/fgene.2012.00250

**Published:** 2012-11-19

**Authors:** Margaret G. Distler, Abraham A. Palmer

**Affiliations:** ^1^Department of Pathology, University of ChicagoChicago, IL, USA; ^2^Department of Human Genetics, University of ChicagoChicago, IL, USA; ^3^Department of Psychiatry and Behavioral Neuroscience, University of ChicagoChicago, IL, USA

**Keywords:** anxiety-like behavior, pain, depression, restless-leg syndrome, GABA, CNV, advanced glycation end-products, AGE

## Abstract

Glyoxalase 1 (GLO1) is a ubiquitous cellular enzyme that participates in the detoxification of methylglyoxal (MG), a cytotoxic byproduct of glycolysis that induces protein modification (advanced glycation end-products, AGEs), oxidative stress, and apoptosis. The concentration of MG is elevated under high-glucose conditions, such as diabetes. As such, GLO1 and MG have been implicated in the pathogenesis of diabetic complications. Recently, findings have linked GLO1 to numerous behavioral phenotypes, including psychiatric diseases (anxiety, depression, schizophrenia, and autism) and pain. This review highlights GLO1's association with behavioral phenotypes, describes recent discoveries that have elucidated the underlying mechanisms, and identifies opportunities for future research.

## Background

Glyoxalase 1 (GLO1) is an enzyme in the glyoxalase system, a metabolic pathway that detoxifies α-oxoaldehydes, particularly methylglyoxal (MG) (Thornalley, 1990, 1993, 2003a; Mannervik, 2008). MG is primarily formed by the degradation of the glycolytic intermediates, dihydroxyacetone phosphate, and glyceraldehyde-3-phosphate (Thornalley, 1993). MG levels rise under high-glucose conditions, such as diabetes (Brownlee, 2001). At supra-physiological levels, MG induces protein and nucleotide modification (advanced glycation end-products, AGEs), reactive oxygen species (ROS), and apoptosis (Brownlee, 2001; Thornalley, 2003b). AGEs cause dysfunction of affected proteins (Brownlee, 2001) and ligate the receptor for advanced glycation end-products (RAGE), triggering the production of ROS (Schleicher and Friess, 2007) and apoptosis (Loh et al., 2006). To combat MG's cytotoxic effects, GLO1 enzymatically converts MG into the less reactive substance, *d*-lactate (Thornalley, 2003b). *In vitro*, overexpression of Glo1 prevents MG accumulation (Shinohara et al., 1998); conversely, GLO1 inhibition increases MG accumulation and decreased cellular viability (Kuhla et al., 2006). Given their roles in AGE formation and cytotoxicity, GLO1 and MG have been implicated in diseases where these effects are relevant to pathogenesis, such as diabetic complications (i.e., micro- and macro-vascular disease), cancer, and aging (Brownlee, 2001; Thornalley, 2003a; Ahmed and Thornalley, 2007; Morcos et al., 2008; Fleming et al., 2010; Thornalley and Rabbani, 2011).

Surprisingly, an increasing number of studies have identified associations between GLO1 and behavioral phenotypes. In mice, several lines of evidence suggest that Glo1 expression is associated with anxiety-like behavior (Hovatta et al., 2005; Reiner-Benaim et al., 2007; Loos et al., 2009; Benton et al., 2011; Distler et al., 2012), depression (Benton et al., 2011), and neuropathic pain (Jack et al., 2011, 2012; Bierhaus et al., 2012). New observations in rats using oxidative stress models (Salim et al., 2010a,b) and the sleep-deprivation model of psychological stress (Vollert et al., 2011), both suggest role of GLO1 in the anxious phenotype of rats (Salim et al., 2011). Additional associations have been identified with habituation, locomotor activity, motor coordination, exploratory behavior, and learning and memory (Williams et al., 2009). In humans, genetic studies have implicated polymorphisms in GLO1 in panic disorder (Politi et al., 2006), depression (Fujimoto et al., 2008), autism (Junaid et al., 2004; Barua et al., 2011), schizophrenia (Arai et al., 2010; Toyosima et al., 2011), and restless legs syndrome (RLS) (Stefansson et al., 2007; Winkelmann et al., 2007, 2011; Kemlink et al., 2009). While the studies of RLS identify a haplotype block that includes GLO1, more attention has been paid to the neighboring gene BTBD9. The other human genetic studies utilized a candidate gene approach and had small sample sizes; in most cases, those results have not been replicated. Aside from genome-wide association studies (GWAS) of RLS, GLO1 has not been identified in GWAS for other psychiatric or neurological traits.

Recently, two major studies have shed light on the mechanisms underlying GLO1's behavioral effects. One identified MG as an agonist at GABA_A_ receptors (Distler et al., 2012), while the other identified MG modification of voltage-gated sodium channels (Bierhaus et al., 2012). This article will focus on GLO1's behavioral correlates and the potential underlying mechanisms. The basic biochemistry of GLO1 has been reviewed elsewhere (Thornalley, 1990, 1993, 2003b), as have its roles in cancer (Thornalley, 2003a; Thornalley and Rabbani, 2011) and diabetic complications (Brownlee, 2001; Ahmed and Thornalley, 2007; Schleicher and Friess, 2007; Jack and Wright, 2012; Rabbani and Thornalley, 2012).

## GLO1's behavioral correlates

### Anxiety

#### Mouse genetic studies

Among the behavioral phenotypes associated with GLO1, anxiety-like behavior is the most commonly reported and widely studied. In mice, numerous genetic studies have identified associations between Glo1 expression and anxiety-like behavior (Hovatta et al., 2005; Kromer et al., 2005; Williams et al., 2009). Hovatta et al. analyzed gene expression profiles in a panel of six inbred mouse strains, which were scored for anxiety-like behavior in the open field and light-dark box tests (Hovatta et al., 2005). They found a positive correlation between Glo1 expression and anxiety-like behavior. Several subsequent studies also identified positive correlations between Glo1 expression and anxiety-like behavior among inbred mouse strains (Reiner-Benaim et al., 2007; Loos et al., 2009; Benton et al., 2011).

Importantly, Hovatta et al. established a causal role for Glo1 in anxiety-like behavior by using viral vectors to overexpress or knock down Glo1 in the anterior cingulate cortex (Hovatta et al., 2005). Local Glo1 overexpression increased anxiety-like behavior, while local Glo1 knockdown decreased anxiety-like behavior (Hovatta et al., 2005). This experimental manipulation supported a causal role for Glo1 in anxiety-like behavior. However, this study was limited by the use of viral vectors: expression was manipulated only in one brain region during adulthood. As such, it might not have accurately modeled Glo1's normal physiological role in anxiety-like behavior. Furthermore, viral vectors often produce variable expression levels between animals (Kirik and Bjorklund, 2003).

Kromer et al. also identified a correlation between GLO1 and anxiety-like behavior in mice (Kromer et al., 2005). They selectively bred mice for high anxiety-like behavior (HAB) or low anxiety-like behavior (LAB) and subsequently inbred the mice to generate two lines. The LAB line had higher GLO1 protein levels than the HAB line (Kromer et al., 2005). Therefore, Kromer et al. proposed that increased GLO1 was a biomarker for low anxiety-like behavior, a result directionally opposite from studies that used panels of inbred mouse strains (Hovatta et al., 2005; Reiner-Benaim et al., 2007; Loos et al., 2009; Benton et al., 2011) and those that used viral vectors to manipulate Glo1 expression (Hovatta et al., 2005). In fact, the discrepancy among these studies was used as grounds to dispute a role for Glo1 in anxiety-like behavior (Thornalley, 2006).

A subsequent study identified a common copy number variant (CNV) among inbred mice that caused a duplication of four genes, including Glo1 (Williams et al., 2009). Of the 72 inbred strains examined, 23 carried the duplication. The duplication was positively correlated with increased Glo1 expression (it was an expression QTL; eQTL) and increased anxiety-like behavior (Williams et al., 2009). Increased Glo1 copy number was also associated with other behavioral phenotypes that were available from public databases, including locomotor habituation in a novel environment (negative correlation), total locomotor activity (positive correlation), motor coordination (positive correlation), rearing (positive correlation), anxiety-like behavior in the elevated plus maze and light-dark box tests (positive correlation), stretch-attends in tests of anxiety-like behavior (negative correlation), and measures of learning and memory (Williams et al., 2009). These findings suggested a role for Glo1 copy number in a broad range of behavioral phenotypes.

The discovery of this CNV helped to explain the previously reported differences in Glo1 expression among inbred strains (Hovatta et al., 2005). In addition, it explained the differential GLO1 protein expression reported in the HAB and LAB lines: LAB mice were subsequently shown to have the duplication, while HAB mice do not (Hambsch et al., 2010). Although it is somewhat surprising that selection did not have the opposite result, other factors, including differences in initial allelic frequencies, linked alleles, and drift before or during inbreeding could have contributed to the fixation of the duplication in LAB but not HAB mice. The effect of the Glo1 duplication on anxiety-like behavior was likely offset by numerous other alleles that collectively contributed to anxiety-like behavior in the selected lines. HAB and LAB lines are known to harbor many genetic differences due to selection and inbreeding (Bunck et al., 2009; Ditzen et al., 2010; Czibere et al., 2011; Filiou et al., 2011; Tasan et al., 2011; Zhang et al., 2011). Indeed, selection studies are prone to such confounding forces, especially when the selected lines are not replicated.

The discovery of the Glo1 CNV also raised the possibility that Glo1 expression could simply be a marker for the presence of the CNV rather than directly regulating anxiety-like behavior. Specifically, the CNV contained four genes, any of which could have affected behavioral phenotypes. Further, the CNV could have been linked to unidentified causal alleles or could have disrupted neighboring genes. As such, the discovery of the CNV complicated observed associations between Glo1 expression and behavior.

Therefore, in order to test the hypothesis that increased Glo1 expression was sufficient to replicated the behavioral differences associated with the CNV, targeted genetic manipulation of Glo1 copy number performed in which mice with a transgenic bacterial artificial chromosome (BAC) containing Glo1 were created to model the CNV (Distler et al., 2012). To isolate Glo1's effect, the BAC was engineered so that only Glo1 was expressed. BAC transgenic mice overexpressed Glo1 and displayed increased anxiety-like behavior. This effect was observed in multiple founder lines and on two genetic backgrounds: C57BL/6J (B6) and FVB/NJ (FVB). The effect of Glo1 overexpression on anxiety-like behavior was strongest in the lines with the highest copy numbers. Unlike the viral vector studies (Hovatta et al., 2005), this study demonstrated the physiological relevance of Glo1 in behavior, because Glo1 was expressed under its endogenous promoter elements, in its natural expression pattern, and throughout the animal's life. Therefore, this study demonstrated that increased Glo1 copy number increased Glo1 expression and anxiety-like behavior.

#### Human genetic studies of GLO1 and anxiety

In contrast to mouse studies, human genetic studies have not identified robust associations between GLO1 and anxiety disorders. In humans, there are two common GLO1 alleles (Kompf et al., 1975) that result from a single nucleotide polymorphism (SNP) at position 419 (Kim et al., 1995). Adenine (419A) encodes a glutamate at amino acid 111 (111E), and cytosine (419C) encodes an alanine (111A). Politi et al. (2006) identified a weak, but significant increase in the risk of panic disorder without agoraphobia among people carrying the 419A allele. Limitations, such as the small sample size and potential population stratification, made this result preliminary. A subsequent study found no correlation between GLO1 expression and susceptibility to panic attacks (Eser et al., 2011), but suffered from the same limitations, so cannot be said to disprove the original observation. Similarly, no published GWAS studies of panic disorder have identified GLO1 (Erhardt et al., 2011). Despite disappointing findings from human genetic studies, there remains a need for large, carefully controlled genetic studies in order to address whether differences in GLO1 affect anxiety among humans.

### Depression

There is strong evidence for shared genetic vulnerability to anxiety and depression (Cerda et al., 2010). Among a panel of inbred mice, Benton et al. found a positive correlation between GLO1 protein levels and baseline depression-like behavior using the tail-suspension test (Benton et al., 2011), suggesting that Glo1 could contribute to the common genetic etiology of anxiety and depression. However, a small human study of GLO1 and depression reported apparently conflicting data. Fujimoto et al. reported a negative correlation between GLO1 expression and depression in humans (Fujimoto et al., 2008). GLO1 mRNA expression was reduced in peripheral white blood cells (WBC) of patients with major depressive and bipolar disorders (Fujimoto et al., 2008). GLO1 mRNA expression did not significantly differ between patients in remission and healthy controls, suggesting that reduced GLO1 expression was a state-dependent marker for depression (Fujimoto et al., 2008). However, this study did not establish whether reduced GLO1 expression affected GLO1 protein levels or enzymatic activity, which would reflect the functional significance of differences in mRNA expression. Further, WBC mRNA may not adequately reflect mRNA expression in the brain (Mehta et al., 2010). Corroborating findings from post-mortem brain samples would significantly strengthen the association between GLO1 expression and depression (Mehta et al., 2010). Therefore, although data from mice implicate Glo1 in increasing depression-like behavior, further experiments are required to resolve the discrepancy with human data.

### Autism

Human studies have proposed an association between a coding SNP in GLO1 (419A/C; rs2736654) and autism. Junaid et al. reported that the 419A allele was 1.5 times more common among autistic patients than controls (Junaid et al., 2004). However, this study was limited by its small sample size and potential confounding variables (e.g., population stratification). Indeed, subsequent studies have not replicated this finding: there was no association between SNPs in GLO1 and autism among Han Chinese patients (Wu et al., 2008), nor was there significant linkage or association between SNPs in GLO1 and autism among Finnish patients (Rehnstrom et al., 2008). Furthermore, a family-based and case-control study found that the 419A allele was more common among unaffected siblings of autistic patients, indicating that it might be protective (Sacco et al., 2007). Finally, several human genome-wide association studies (GWAS) have failed to identify associations between GLO1 polymorphisms and autism. At the very least, this suggests that the study of Junaid et al. overestimated the effect size of this allele, which must have been small enough to escape detection by larger GWAS.

Nevertheless, Junaid et al. suggested that the 419A GLO1 allele was associated with a functional change in GLO1 enzymatic activity. They reported that post-mortem brain tissue from autistic patients had reduced GLO1 enzymatic activity and increased AGE content compared to that from control patients (Junaid et al., 2004). In a later study, Baura et al. reported a correlation between the 111E GLO1 isoform and reduced enzymatic activity (Barua et al., 2011). However, Barua et al. did not directly examine enzymatic activity of the isoforms. Rather, they utilized cell extracts derived from autistic and control patients. As such, differences in enzymatic activity and MG concentration could have resulted from differences in GLO1 expression or other genetic differences. Therefore, further studies will help determine the functional significance of the 419A/C SNP and its contribution to autism.

Experimental studies in mice may help determine whether GLO1 is related to autism-like behaviors. Various behavioral assays have been proposed for measuring autism-like phenotypes in mice, including social interaction tests, ultrasonic vocalization measurements, assessment of repetitive and stereotyped behavior, and reversal learning tests (Silverman et al., 2010). In future studies, these assays may be used to study mutant mice with increased or decreased Glo1 expression.

### Schizophrenia

Recently, two studies have suggested a role for GLO1 in schizophrenia (Arai et al., 2010; Toyosima et al., 2011). In a single schizophrenic patient, a frameshift mutation in GLO1 was correlated with reduced GLO1 enzymatic activity (Toyosima et al., 2011). In a separate study, schizophrenic patients were found to have increased AGE accumulation compared to control subjects (Arai et al., 2010), suggesting reduced GLO1 function. Nevertheless, small sample sizes and confounding variables made these associations preliminary. Therefore, additional human genetic studies and studies of GLO1 in mice will help establish an association with schizophrenia. Several behavioral assays of schizophrenia-like behavior have been proposed (Young et al., 2010), which could be used for studying mice with aberrant Glo1 expression.

### Restless legs syndrome

Multiple genome-wide association studies have implicated a haplotype block that includes GLO1 in restless legs syndrome (Stefansson et al., 2007; Winkelmann et al., 2007, 2011; Kemlink et al., 2009). These studies have focused on BTBD9, another gene that, like GLO1, is in this largely non-recombinant haplotype, but this association could also reflect regulatory changes that impact GLO1.

### Pain

Neuropathic pain is a feature of neuropathy, a common sequela of diabetes (Vinik et al., 2000; Edwards et al., 2008). There is an extensive literature on the relationship between Glo1 and neuropathy which has been recently reviewed (Jack and Wright, 2012). Recent studies have identified associations between the Glo1 CNV in mice and behavioral measures of neuropathic pain (Jack et al., 2011, 2012). After diabetes induction, A/J mice, which carry a triplication of the Glo1 allele, were less sensitive to mechanical pain than B6 mice, which have only a single copy of Glo1 (Jack et al., 2011). Similarly, Glo1 copy number was associated with mechanical pain sensitivity after induction of diabetes in two closely related inbred strains, BALB/cJ and BALB/cByJ. BALB/cByJ mice carry the Glo1 duplication, while BALB/cJ mice do not (Williams et al., 2009). Similar to the previous report, increased levels of Glo1 protected against diabetic hyperalgesia in BALB/cByJ mice compared to BALB/cJ mice (Jack et al., 2012). These results suggested that Glo1 mediates behavioral responses to pain associated with diabetic neuropathy. A recent study has confirmed that GLO1 protects against neuropathic pain associated with diabetes. Overexpression of human GLO1 reduced thermal hyperalgesia in diabetic mice (Bierhaus et al., 2012). Conversely, pharmacological inhibition of GLO1 and Glo1 knockdown exacerbated both mechanical and thermal hyperalgesia in diabetic mice (Bierhaus et al., 2012). Together, these findings suggest a role for GLO1 in mediating behavioral measures of pain, particularly that related to diabetic neuropathy. To date, no study has investigated the role of GLO1 in other types of pain, such as nociceptive and central pain, which is an important area of future investigation.

## GLO1's mechanism of action in behavior

### MG regulates behavior

There is robust evidence that GLO1 activity regulates MG concentration (Thornalley, 1990, 1993, 2003b). Recent studies have suggested this activity is critical to GLO1's effects on behavior. *In vitro*, Glo1 overexpression prevented MG accumulation under conditions of high glucose (Shinohara et al., 1998), and in C. elegans, Glo1 overexpression reduced basal MG concentration (Morcos et al., 2008). Similarly, Glo1 overexpression reduced baseline MG concentration in the brains of mice (Distler et al., 2012) and plasma MG concentration in diabetic mice (Bierhaus et al., 2012).

Given the strong association between GLO1 and MG concentration, it was hypothesized that GLO1 affects behavior by controlling levels of MG. This hypothesis suggested that direct administration of MG would alter behavior. Recent reports have demonstrated MG's behavioral effects, including those related to anxiety-like behavior (Hambsch et al., 2010; Distler et al., 2012), depression (Zhang et al., 2011), motor coordination (Distler et al., 2012), and pain (Bierhaus et al., 2012). Hambsch et al. administered MG to mice by intra-cereberoventricular injection daily for 6 days; MG-treated mice displayed reduced anxiety-like behavior in the elevated plus maze compared to vehicle-treated mice (Hambsch et al., 2010). Chronic MG treatment also reduced immobility in the tail suspension test, reflecting an anti-depressant effect of MG (Hambsch et al., 2010). Distler et al. identified a similar anxiolytic effect of MG within minutes of a single intra-peritoneal injection (Distler et al., 2012), suggesting that MG acutely affects behavior. At high doses, acute MG administration was also shown to have sedative effects, including locomotor depression, ataxia, hypothermia, and lethargy (Distler et al., 2012). Acute MG administration also caused hyperalgesia equivalent to that caused by diabetes in mice (Bierhaus et al., 2012). Together, these studies indicated that MG concentration regulates multiple behavioral phenotypes and that MG's effects are opposite to those of Glo1 overexpression.

### Behavioral effects of pure MG

The behavioral experiments described above utilized crude MG preparations, which may contain contaminants, such as formaldehyde and methanol (Pourmotabbed and Creighton, 1986; McLellan and Thornalley, 1992). We recently tested the behavioral effects of pure MG and found that it elicited similar pharmacological effects as crude MG. At a low dose, pure MG was anxiolytic in the open field test, and at a high dose, it caused locomotor depression, ataxia, and hypothermia (Distler et al., 2012). Notably, the doses of pure MG required for sedation, locomotor depression, ataxia, and hypothermia were similar to the doses of crude MG, while pure MG elicited an anxiolytic effect at a lower dose than crude MG. It remains to be determined whether pure MG affects other reported behavioral effects, such as depression and hyperalgesia.

### MG's mechanism of action in behavior: age formation

MG is best characterized as a cytotoxic agent that forms AGEs. This molecular action has been attributed to the pathogenesis of several diseases characterized by increased MG concentrations, including diabetic complications. For instance, increased MG levels and subsequent AGE formation have been well-established pathogenic mechanisms in diabetes-associated microvascular disease (e.g., diabetic retinopathy) and macrovascular disease (e.g., atherosclerosis) (Brownlee, 2001).

Recently, AGEs have been attributed to the development of diabetic neuropathy and neuropathic pain (Jack and Wright, 2012). Induction of diabetes in rats increased AGEs in the extracellular matrix of sensory nerves (Duran-Jimenez et al., 2009). In particular, laminin and fibronectin were found to be modified by MG (Duran-Jimenez et al., 2009). Among the numerous proteins likely modified by MG in diabetes (Rabbani and Thornalley, 2012), a voltage-gated sodium channel, Na_v_1.8, was recently reported to be modified by MG and it was suggested that this modification contribute to neuropathic pain (Bierhaus et al., 2012). Na_v_1.8 was MG-modified in peripheral sensory neurons of mice subjected to diabetes, MG administration, or Glo1 knock down. Knock down of Na_v_1.8 reduced MG-induced thermal hyperalgesia. Finally, MG-modification of Na_v_1.8 dysregulated electrical signaling in sensory neurons. Specifically, prolonged incubation with MG (3–14 h) modified Na_v_1.8 and altered resting membrane potential, current threshold, and voltage threshold. These findings suggested that AGEs promote protein dysfunction under conditions of high MG concentrations, thus contributing to neuropathic pain.

Because MG's role in AGE formation is well characterized, it was thought that this mechanism was relevant to other behavioral phenotypes, including anxiety-like behavior and depression (Hambsch, 2011). Hambsch et al. proposed that MG-dependent AGE formation contributed to MG's anxiolytic action (Thornalley, 2006; Hambsch et al., 2010). They found increased AGEs in the brains of mice treated chronically with MG by intracerebroventricular administration. Hambsch et al. utilized a high dose of MG, corresponding to an average concentration of 1.6 mM throughout the brain (assuming a brain volume of 0.45 mL). This study did not establish a causal relationship between AGE formation and anxiety-like behavior. Therefore, a role for AGE formation in anxiety-like behavior remains uncertain. Further, the discovery that MG activates GABA_A_ receptors (discussed below) provides a compelling alternative mechanism for MG's anxiolytic effect.

### MG's mechanism of action in behavior: GABA_A_ receptor activation

A novel role for MG-independent of AGE formation was recently identified. MG was found to activate GABA_A_ receptors at physiological concentrations (Distler et al., 2012). *In vivo*, MG's pharmacodynamic profile was similar to that of known GABA_A_ receptor agonists, which are anxiolytic at low doses and have sedative effects at higher doses (Rudolph and Mohler, 2004; Carter et al., 2009; Kumar et al., 2009). *In vitro*, MG selectively activated GABA_A_ receptors and was characterized as a competitive partial agonist (Distler et al., 2012). There is a wealth of evidence that GABA_A_ receptors play key roles in anxiety (Kent et al., 2002; Kalueff and Nutt, 2007). As such, MG's activity at GABA_A_ receptors can account its anxiolytic effect.

This mechanism is distinct from MG's previously reported cellular effects, specifically AGE formation and cytotoxicity. A low dose of MG was anxiolytic within minutes of peripheral administration (Distler et al., 2012). This time course differs from that of AGE formation, which requires hours to days (Lo et al., 1994). Therefore, AGE formation is unlikely to mediate MG's effect on anxiety-like behavior. MG's anxiolytic effect was also independent of cytotoxicity. *In vitro*, MG concentrations of 100–1000 μM have been reported to induce neuronal cell death (Di Loreto et al., 2004, 2008; Li et al., 2010). In contrast, an anxiolytic dose of MG only increased the concentration of MG in the brain to approximately 6 μM (Distler et al., 2012). In addition, MG requires hours to days to induce apoptosis (Di Loreto et al., 2008), which is inconsistent with the acute time course (~10 min) of MG's behavioral effects. Finally, there was no evidence of apoptosis in mice treated with an anxiolytic dose of MG (Distler et al., 2012). Therefore, MG's effect at GABA_A_ receptors occurs at lower concentrations and is temporally dissociable for its better-known effects, such as AGE formation and cytotoxicity. MG's GABAergic action may explain previous observations of MG's electrophysiological effects. For example, Hambsch et al. found that incubation with 10 μM MG reduced the magnitude of long-term potentiation (LTP) in hippocampal brain slices (Hambsch et al., 2010). This is characteristic of GABA_A_ receptor activation, which decreases excitatory post-synaptic potentials and suppresses LTP in CA1 slices (Seabrook et al., 1997).

### Implications of MG's activity at GABA_A_ receptors

**Figure 1:**
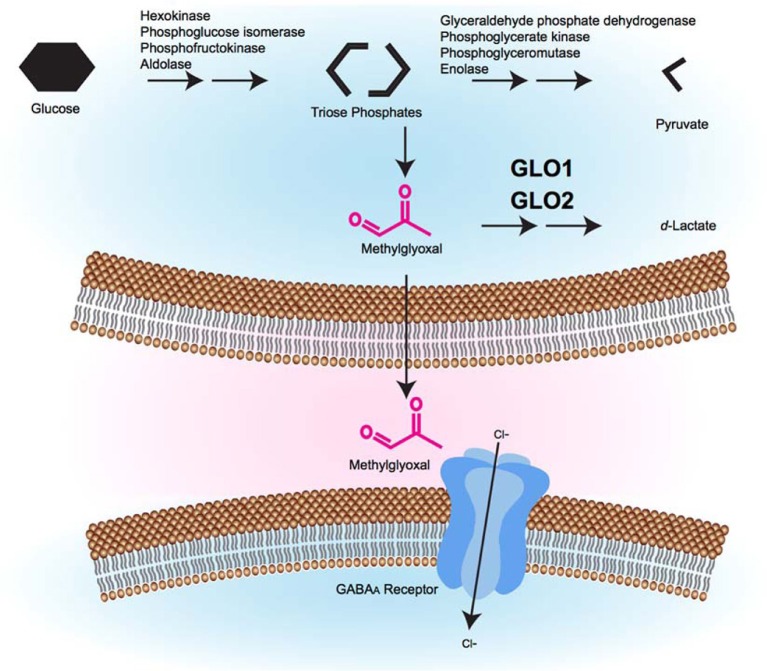
**A model of the glyoxalase system's role in GABAergic signaling.** During glycolysis, triose phosphate intermediates can be converted to MG by non-enzymatic fragmentation. Excess MG is catabolized by the glyoxalase system (GLO1 and GLO2) to form *d*-lactate. MG that is not catabolized can cross the plasma membrane, where it accesses pre- or post-synaptic GABA_A_ receptors where it causes GABA_A_ receptor activation, inward Cl^−^ current, and membrane hyperpolarization, which is hypothesized to alter behavior.

MG's role in activating GABA_A_ receptors has important physiological implications. MG production increases during high glycolytic activity. MG can then be metabolized by GLO1, can form AGEs or can activate GABA_A_ receptors (Figure 1). Importantly, the effects at GABA_A_ receptors occur at physiological concentrations of MG. Similarly, changes in MG concentration that are associated with diabetes can lead to changes in AGE formation at a variety of targets, including Nav1.8, which appears to modulate pain sensitivity.

One physiological function of MG's activity at GABA_A_ receptors may be to maintain inhibitory tone under conditions of low GABA concentrations. In the synaptic cleft, the concentration of GABA is in the millimolar range during synaptic release (Farrant and Nusser, 2005); in the extracellular space, GABA concentrations are in the submicromolar range (Vithlani et al., 2010). The concentration of MG in the brain is approximately 5 μM (Distler et al., 2012), and these levels are likely similar in both the synapse and extracellular space. Therefore, MG is less abundant than GABA at the synapse; however, in the extracellular space MG is more abundant than GABA and likely acts as a partial agonist (Distler et al., 2012). Thus, while it has yet to be well explored MG may have a complex role in the regulation of GABAergic signaling.

While there are no data to directly support the possibility, another physiological role for MG may be as a negative regulator of excitatory signaling. Excitatory synaptic transmission is associated with increased glycolysis (Barros and Deitmer, 2010). In fact, 60–75% of neuronal glucose utilization is estimated to be in glutamatergic neurons (Shulman et al., 2004). Since neuronal excitation is coupled with glycolysis, MG concentrations may increase in excitatory neurons. MG would then activate local GABA_A_ receptors and thus serve as a negative feedback signal. In addition, it would also provide inhibitory tone to neighboring cells. Such an action could be neuroprotective, since excessive N-methyl-D-aspartate (NMDA) receptor activation by glutamate triggers excitotoxicity and apoptosis (Naegele, 2007; Henshall and Murphy, 2008).

At the organismal level, MG concentration increases under conditions of high glucose load. Accordingly, it is possible that MG concentration may link metabolic state to neuronal inhibitory tone and behavior, including anxiety. Specifically, high levels of glucose may be coupled to reduced anxiety through increased levels of MG. In humans, consumption of foods high in fat and sugar is associated with emotional reward (Jacquier et al., 2012). In rats, fructose consumption increased levels of MG in rats (Wang et al., 2008), which could provide a direct link between the intake of high-sugar foods and emotional satiety. One notable exception to the association between increased metabolic load and reduced anxiety may be in diabetic patients, where elevated MG level have consistently been observed (Shinohara et al., 1998; Brownlee, 2001; Masterjohn et al., 2011). There is an increased prevalence of anxiety among diabetic patients (Anderson et al., 2002), whereas higher concentrations of MG would be expected to decrease anxiety. Nevertheless, it remains possible that diabetic patients experience increased anxiety in spite of increased MG concentration, since diabetes is clearly associated with numerous physiological changes in addition to changes in MG concentration and is correlated with numerous environmental and epidemiological factors. Thus, the presence of increased anxiety in diabetes patients is difficult to interpret.

## Future directions

### GLO1's effects in females

Glo1's effect on anxiety-like behavior has not been assessed in females. Virtually all of the studies that identified associations between Glo1 expression and anxiety-like behavior utilized only male mice (Hovatta et al., 2005; Loos et al., 2009; Benton et al., 2011), although there are isolated exceptions (Williams et al., 2009). Studies that directly manipulated Glo1 overexpression by viral vectors (Hovatta et al., 2005) and BAC transgenes (Distler et al., 2012) studied behavioral effects in male mice only. It would not be unprecedented if the behavioral effects of Glo1 are sex-specific; sex differences in anxiety have been described in both humans and animal models (Palanza, 2001; Hamann, 2005; McLean and Anderson, 2009). The etiology underlying differential anxiety between males and females is not well understood, but it likely includes environmental and hormonal factors (McLean and Anderson, 2009; Solomon and Herman, 2009; Nillni et al., 2011).

### GLO1 copy number and anxiety-like behavior in mice

Another unresolved issue is that only the BAC transgenic lines with high Glo1 copy numbers, and correspondingly high Glo1 expression, exhibited increased anxiety-like behavior (Distler et al., 2012). The initial association between Glo1 copy number and anxiety-like behavior involved a duplication. In contrast, B6 and FVB BAC transgenic mice with two additional copies of Glo1 (equivalent to a homozygous duplication) did not significantly differ from wild-type mice in anxiety-like behavior (Distler et al., 2012). This negative result could reflect inadequate power to detect a difference or could reflect a true lack of effect of a Glo1 duplication on anxiety-like behavior. The latter possibility could be due to differences in Glo1 expression despite equal copy numbers at the genomic level. Another possibility is that Glo1 overexpression has more robust anxiogenic effects on specific genetic backgrounds. Consistent with this explanation, Hovatta et al. reported that viral-vector-mediated Glo1 overexpression robustly increased anxiety-like behavior in 129S6/SvEvTac mice but not B6 mice (Hovatta et al., 2005).

### Evolutionary significance

The evolutionary significance of the Glo1 CNV is unknown. Glo1 CNVs are present in both laboratory and wild -caught mice, indicating that the duplicated allele arose prior to the domestication of mice by mouse fanciers and later scientists (Williams et al., 2009). While Redon et al. (2006) reported CNVs that included GLO1 in humans, the underlying data for those particular CNVs are unconvincing. We are not aware of any other evidence that GLO1 CNVs are common among humans or any other species; although it is possible they have simply escaped detection.

MG is byproduct of glycolysis that is generated in every organism. Homologs of GLO1 exist in bacteria, fungi, plants, and animals (Cooper, 1984; Yadav et al., 2008; Inoue et al., 2011), highlighting its ancient physiological importance. Because MG accumulation is cytotoxic, GLO1's conservation is likely tied to its role in cellular survival. The extent to which MG's action at GABA_A_ receptors is conserved is unknown. GABA exists in bacteria, fungi, plants, and animals (Guthrie and Nicholson-Guthrie, 1989; Rauh et al., 1990; Kumar and Punekar, 1997; Bouche and Fromm, 2004), though it is only thought to serve as a neurotransmitter among animals; GABA_A_ receptors are prominent among animals, including invertebrates (Rauh et al., 1990). Therefore, an important future direction will be to define the evolution of the relationship between MG and GABA_A_ receptors in other species. It is possible that MG acts at GABA_A_ receptors in invertebrates; application of 10–150 μM MG had an excitatory effect on the sixth abdominal ganglion of the cockroach (Davies et al., 1986). Although GABA is an inhibitory neurotransmitter in insects, it is also excitatory in some cell types (Beg and Jorgensen, 2003; Gisselmann et al., 2004).

### MG's biophysical interaction with GABA_A_ receptors

**Figure 2:**
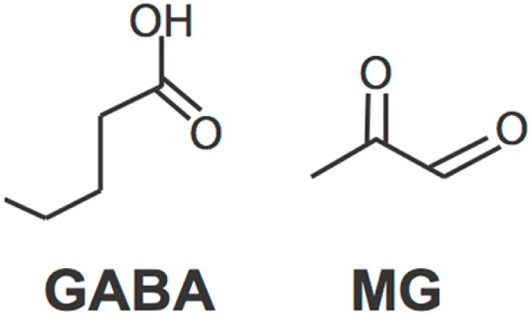
**Molecular structures of GABA and MG**.

The discovery that MG activates GABA_A_ receptors has created an exciting new direction of research. Specifically, studying MG's biophysiocal interactions with GABA_A_ receptors is liable to yield important results. Although MG and GABA are both small molecules, they are not structurally similar (Figure 2). Therefore, it is unknown whether they share a binding site on GABA_A_ receptors or whether binding of one prevents binding of the other. Structural studies are necessary to resolve this question.

Similarly, GABA_A_ receptors are pentamers comprised of various combinations of at least 16 possible subunits: α1–6, β1–3, γ1–3, δ, ε, θ, and π (Bollan et al., 2003). Future studies will be required to identify MG's receptor subtype selectivity. This is relevant to MG's behavioral effects, because different subunits and subunit compositions mediate different GABAergic effects. For instance, α2 subunits have been reported to mediate the anxiolytic effects of benzodiazepines, and α5 subunits are thought to mediate their sedative effects (Rudolph and Knoflach, 2011). MG's activity at different receptor subtypes may contribute to its endogenous role in the CNS. Future studies will be critical in identifying the pharmacological and biophysical responses of specific GABA_A_ receptor compositions to MG.

Future studies will also be important for assessing MG's activity at synaptic and extra-synaptic GABA_A_ receptors. Synaptic GABA_A_ receptors regulate phasic inhibitory signaling, while extra-synaptic GABA_A_ receptors contribute to tonic inhibitory signaling (Hablitz et al., 2009) and anxiety (Maguire et al., 2005). Future studies must directly measure MG's activity at synaptic and extra-synaptic receptors in order to determine MG's role in phasic and tonic inhibitory signaling.

### GLO1's mechanism of action in other behavioral phenotypes

As reviewed above, two major mechanisms have been ascribed to GLO1's behavioral effects: regulating AGE formation and GABA_A_ receptor activation. These mechanisms are proposed to mediate GLO1's role in pain and anxiety, respectively. However, the extent to which each mechanism underlies the many other behavioral correlates of GLO1 remains an important area of future investigation. For instance, it is possible that GLO1 regulates depression through its role in modulating GABAergic tone, given evidence that deficits in the GABA system have been implicated in depression (Gerner and Hare, 1981; Petty and Schlesser, 1981; Petty and Sherman, 1984; Crestani et al., 1999; Earnheart et al., 2007; Shen et al., 2010; Hasler and Northoff, 2011; Luscher et al., 2011). Similarly, the mechanism underlying GLO1's putative involvement in autism remains unknown. Autism has been associated with high levels of MG and AGEs (Junaid et al., 2004); however, few studies have examined whether AGEs contribute to autism (Boso et al., 2006). Alternatively, it is possible that GLO1's role in GABAergic signaling could contribute to autism since disruptions in GABAergic signaling have been identified in autism (Di Cristo, 2007; Rubenstein, 2011). Therefore, additional studies in humans and mice are required to elucidate the mechanisms underlying GLO1's associations with additional behavioral phenotypes.

### GLO1 as a target for therapeutic agents

GLO1 may be a useful target for pharmacological intervention. For instance, GLO1 inhibition was shown to increase MG concentration and reduce anxiety-like behavior *in vivo* (Distler et al., 2012). GLO1 inhibition would represent a novel mechanism of action among psychiatric drugs. Most current anxiolytic therapies target neurotransmitter systems, including the serotonin and GABA systems (Pollack, 2009). In contrast, GLO1 inhibitors would increase the production of MG, an endogenous GABA_A_ receptor agonist. This could have a different and possibly more favorable side-effect profile compared to agents that allosterically activate GABA_A_ receptors. For instance, endogenous MG might have receptor subtype specificity, thus preventing off-target effects. Similarly, GLO1 inhibition may not increase MG levels sufficiently to cause sedation or have abuse potential, which are common concerns of current GABA_A_ receptor agonists. Development of better GLO1 inhibitors and characterization of the side-effects of GLO1 inhibition are thus important goals for future studies.

Studies of the adverse effects of GLO1 inhibition *in vivo* must also pay particular attention to cytotoxicity. *In vitro* studies have demonstrated that GLO1 inhibition can cause cell death (Kuhla et al., 2006). Over a short period of time, this is unlikely to occur *in vivo*, since GLO1 inhibition was shown to only modestly increase MG levels. Nevertheless, chronic GLO1 inhibition may increase MG to more toxic levels, causing AGE formation or cell death. Since AGEs play a prominent role in diabetic complications, GLO1 inhibitors may be a poor choice for patients with diabetes (Brownlee, 2001). In particular, GLO1 inhibition was recently shown to exacerbate diabetic hyperalgesia (Bierhaus et al., 2012), which could limit the use of GLO1 inhibitors, especially in diabetic patients.

## Conclusion

Despite a controversial history, recent studies have demonstrated roles for GLO1 in behavioral phenotypes, including anxiety-like behavior and pain. Although animal studies support a role for GLO1 in behavioral phenotypes, human genetic studies are less convincing. As such, the extent to which polymorphisms in GLO1 regulate human diseases remains an important direction for future studies. MG can induce protein modification and acts as an endogenous GABA_A_ receptor agonist. These cellular functions likely contribute to MG's behavioral effects. GLO1 inhibitors may provide novel therapeutic tools for the treatment of CNS disorders.

### Conflict of interest statement

The authors declare that the research was conducted in the absence of any commercial or financial relationships that could be construed as a potential conflict of interest.
